# The microbicidal potential of visible blue light in clinical medicine and public health

**DOI:** 10.3389/fmed.2022.905606

**Published:** 2022-07-22

**Authors:** Devika Haridas, Chintamani D. Atreya

**Affiliations:** ^1^School of Medicine, University of Maryland, Baltimore, Baltimore, MD, United States; ^2^Laboratory of Cellular Hematology, Division of Blood Components and Devices, Office of Blood Research and Review, Center for Biologics Evaluation and Research, Food and Drug Administration, Bethesda, MD, United States

**Keywords:** violet-blue light, antimicrobial, pathogen reduction, biofilm, microbes

## Abstract

Visible blue light of wavelengths in the 400–470 nm range has been observed to have microbicidal properties. A widely accepted hypothesis for the mechanism of microbial inactivation by visible blue light is that the light causes photoexcitation of either endogenous (present within the microbe) or, exogenous (present in the biological medium surrounding the microbe) photosensitizers such as porphyrins and flavins, which leads to the release of reactive oxygen species that subsequently manifests microbicidal activity. Some of the factors that have been observed to be associated with enhanced microbicidal action include increased duration of exposure, and either pre- or co-treatment with quinine hydrochloride. In case of bacteria, repetitive exposure to the blue light shows no significant evidence of resistance development. Additionally, visible blue light has exhibited the ability to inactivate fungal and viral pathogens and, multidrug-resistant bacteria as well as bacterial biofilms. Visible blue light has demonstrated efficacy in eliminating foodborne pathogens found on food surfaces and exposed surfaces in the food processing environment as well as in the decontamination of surfaces in the clinical environment to minimize the spread of nosocomial infections. We conclude from reviewing existing literature on the application of the blue light in clinical medicine and public health settings that this microbicidal light is emerging as a safer alternative to conventional ultraviolet light-based technologies in multiple settings. However, further comprehensive studies and thorough understanding of the mechanism of microbicidal action of this light in different scenarios is warranted to determine its place in human health and disease.

## Introduction

The purpose of this literature review is to provide a comprehensive overview of the potential of the blue light as a microbicidal tool by examining its clinical and public health applications as evidenced by experimental studies published in the last 10 years. Additional areas of investigation within this literature review will include the mechanism of pathogen inactivation by the blue light, conditions that optimize its microbicidal activity, its efficacy on different types and forms of pathogens, and its effects on normal healthy host (mammalian) cells.

The omnipresence of different forms of pathogens poses a multitude of risks to health of the individuals and population at large. For this reason, the technologies that reduce or inactivate pathogens have applications in food safety, environmental health, and clinical contexts. Emerging technologies for pathogen reduction can also have far-reaching public health consequences, with implications for long-standing issues like antibiotic-resistant bacteria, and the incidence of hospital-acquired infections. One such emerging technology is the application of visible blue light of wavelengths in the 400–470 nm range, hitherto referred as “the blue light” throughout this report, is increasingly being investigated for its microbicidal properties against a variety of pathogens in various settings (1, 2). Potential clinical applications of these technologies include the treatment of localized infections and wound healing (2, 3). The blue light also shows potential for facilitating a solution for the increasingly urgent public health concern of antibiotic resistance, as experiments involving repeated exposure to high intensities of blue light have shown no significant evidence of bacterial resistance (4–6). Environmental decontamination applications of the blue light include disinfection of food, contact surfaces, and surrounding air. Foodborne pathogens are estimated to cause annually 9.4 million cases of illness in the United States (7). Among the leading pathogens of these foodborne illnesses are norovirus and non-typhoidal *Salmonella* (7). Microbial persistence and growth on surfaces are another major health concern, particularly in the hospital settings (8). Dry hospital surfaces have been observed to develop biofilms of microbes, which are then less susceptible to conventional disinfection methods (8).

## Mechanism of pathogen inactivation by the blue light

A widely accepted hypothesis regarding the mechanism of photoinactivation of organisms by the blue light is that it causes photoexcitation of endogenous photosensitizers such as flavins and porphyrins, which triggers the release of reactive oxygen species, and in turn leads to oxidative stress and cell toxicity; this was well described in Ramakrishnan et al. (9). This study made several observations using *Staphylococcus epidermidis* and 405 nm light as a model for photoinactivation of bacteria: (a) treatment resulted an overproduction of reactive oxygen species in the treated cells (b) upon addition of a reactive oxygen species (free H_2_O_2_) scavenger sodium pyruvate, to the light-treated *S. epidermidis* cells, the bacterial inactivation was substantially reduced when compared to light-treated *S. epidermidis* cells without sodium pyruvate, (c) greater reduction in *S. epidermidis* inactivation observed when a combination of three reactive oxygen species scavengers (sodium pyruvate, catalase, and dimethyl thiourea) was administered to light-treated cells. These observations lend credence to the fact that more than one reactive oxygen species takes part in the photoinactivation mechanism induced by 405 nm light (9).

A recent study investigated the reason for the known discrepancies in bacterial inactivation by different light wavelengths within the 400–470 nm spectra, with the understanding that differences in the extent of microbial inactivation are typically explained by the presence of different photosensitizers present within the targeted microbe (1). Investigators studied the apparent contradiction in existing knowledge which indicates that 400–420 nm light is more efficient at bacterial inactivation than 450–470 nm light, even for *Enterococci*, which possess flavins but not porphyrins that would predispose them to be more vulnerable to 400–420 nm light (1). Upon exposure of *E. moraviensis* to 405 and 450 nm light, it was found that 405 nm light exposure led to a greater reduction in the bacterial population (1), consistent with findings in prior publications. Fluorescent spectral analysis of *E. moraviensis* exposed to 405 nm light showed decreasing endogenous flavin levels with increasing duration, as well as increasing levels of hydrogenated nicotinamide adenine dinucleotide (NADH) and the fluorophore lumichrome in the bacteria (1). Based on this observation, investigators proposed that although both NADH and lumichrome were increased with increased light dose, the latter could possibly have a role in the efficient inactivation of *E. moraviensis* by 405 nm light, as it is known to have high absorption at 405 nm and is an efficient photosensitizer (1).

Another study elucidated the microbicidal mechanism of the blue light on *Neisseria gonorrhoeae* (3). Upon examination of the fluorescence spectra and absorption spectra of endogenous photosensitizers derived from the *N. gonorrhoeae* cell lysates, presence of both porphyrins and flavins were identified; spectroscopic and ultra-performance liquid chromatography analysis showed that exposure to 405 nm light led to the excitation of porphyrins and flavins and subsequent cell death (3). This study also examined the microbicidal effect of 405 nm light under the hypoxic condition by deoxygenating the *N. gonorrhoeae* cell suspension with N2 prior to the light exposure. It was found that the microbicidal effect of 405 nm was substantially diminished in the hypoxic condition compared to the non-hypoxic control, with only 1-log_10_ reduction and a complete inactivation of the microbe in the non-hypoxic control condition; the fact that there was still some level of bacterial reduction in the hypoxic condition led investigators to infer the involvement of reactive oxygen species (ROS) besides singlet molecular oxygen (1O_2_) in the photoinactivation mechanism, which in turn suggests that flavins that produce ROS have a role in the photosensitization reaction and subsequent photoinactivation of *N. gonorrhoeae* by 405 nm light (3).

## Conditions that optimize microbicidal activity of the blue light

A common finding among studies examining photoinactivation of bacteria upon administration of the blue light is that 405-nm light is the most effective at bacterial reduction, compared to other wavelengths within the 400–470 nm range (1, 3, 10). A study comparing the efficacy of photoinactivation of *Listeria monocytogenes* by blue light wavelengths ranging from 400 to 450-nm in 10-nm increments observed 405 ± 5 nm to be the most efficacious (10). As previously mentioned, it was found in the study by Hessling et al. sthat administration of 405 nm light led to a greater reduction in the bacterial concentration of *Enterococus moraviensis* than did administration of 450 nm light (1). Similarly, Wang et al. study examining the role of wavelength on inactivation of *Neisseria gonorrhoeae* found that 405-nm light led to greater reduction in bacterial concentration than did 470 nm light (3).

McKenzie et al. used 405 nm light in conjunction with different stress conditions to identify the stress conditions that most enhance the bactericidal activity of 405 nm light (11); in these experiments *Escherichia coli* and *Listeria monocytogenes* were subjected to sub-lethal stress conditions including varying temperatures, pH levels, and salt concentrations. After being held in the sub-lethal stress conditions, the bacterial suspensions were exposed to 405 nm light at an irradiance of 70 mW/cm^2^ (11). Temperature stress was observed to induce enhanced photoinactivation with 405-nm light in both species, with the populations grown in the 4° and 45°C stress conditions exhibiting greater inactivation rates than those grown in the non-stress 22°C condition (11). Acid stress was observed to be associated with the most substantial enhancement of photoinactivation (11). After being held in the pH 3 condition, the dose of 405 nm light required to achieve the same extent of inactivation as observed in the non-stressed population was reduced by 77% for *E. coli* and by 50% for *L. monocytogenes* (11). Increased salt concentration produced differential effects on the two species, with *E. coli* requiring a 50% lesser dose for inactivation, whereas *L. monocytogenes* required a 50% higher dose for inactivation (11). Upon further examination, the apparent increase in 405 nm light tolerance in *L. monocytogenes* was explained by the fact that the increased salt condition did not induce sub-lethal damage in *L. monocytogenes* as it did for *E. coli*, as *L. monocytogenes* is known to have a degree of osmo-tolerance (11). Bache et al. comparing the extent of bacterial reduction after different durations of visible blue light treatment, it was found that increased duration of exposure was associated with greater extent of bacterial reduction (12).

## Bacteria do not develop resistance and drug-resistant bacteria show susceptibility to the blue light

Drug-resistance in bacteria is an increasingly important issue that poses a substantial threat to public health on the global scale, as multidrug-resistant bacteria continue to emerge (13, 14). Infectious agents are a major contributor to the global burden of disease and are associated with more than 25% of annual deaths worldwide (13, 15). The development of drug resistance in bacteria limits the prevention and treatment options, and it is therefore of significant consequence to determine both the potential for bacteria to develop resistance to inactivation by the blue light, and the efficacy with which the light can inactivate drug-resistant bacteria. Multiple studies sought to determine whether bacteria are likely to develop resistance to photoinactivation by the blue light and observed no signs of the development of resistance in bacterial samples subjected to repetitive exposures of sublethal light doses (4–6, 16, 17). A 2019 study assessed whether *N. gonorrhoeae* cells could develop resistance to the 405 nm blue light by exposing the bacterial suspension to a subtherapeutic amount of the light, growing the surviving cells in culture overnight, exposing the new suspension to 405 nm light, and repeating this cycle for 15 times (6). Analysis of the bacterial reduction after each successive round of 405 nm light exposure showed no statistically significant association between the number of exposures and extent of bacterial reduction, suggesting that *N. gonorrhoeae* is not inclined to develop resistance to 405 nm antimicrobial blue light (6).

A 2017 study reached a similar conclusion after examining the propensity of *Staphylococcus aureus* to develop resistance to 405 nm light exposure (5). Tomb et al. study conducted one experiment in which *S. aureus* was cultured under low-level exposure to 405 nm light, at an irradiance level of 1 mW/cm^2^ (5). In the second experiment, *S. aureus* was subjected to 15 successive cycles of exposure to a sublethal dose of 108 J/cm^2^ of high-intensity 405 nm light (5). The *S. aureus* cultured under low-level light exposure was observed to require a greater dose of 405 nm light for complete inactivation (5). Upon the subsequent observation of elevated H_2_O_2_ in the *S. aureus* cultured in low-level 405 nm light, it was suggested that the apparent tolerance of these cells to inactivation by 405 nm light may be attributable to the upregulation of protective enzymes induced by the stress of low-level 405 nm light exposure during culture (5). This finding suggests that to avoid engendering bacterial tolerance to 405 nm light in clinical applications, 405 nm light should be administered at a bactericidal dose as opposed to a low-level exposure for longer times (5). In the experiment subjecting *S. aureus* to 15 successive cycles of high-intensity 405 nm light at a sub-lethal dose, there was no significant evidence of the development of tolerance to inactivation by 405 nm light (5). The observation that bacteria do not appear to develop resistance to blue light after repetitive exposure was also supported by the findings of Zhang et al. which subjected multidrug-resistant *A. baumannii* to 10 successive cycles of sublethal exposure to 415 nm light and observed no development of resistance (17). Leanse et al. concluded that there was no statistically significant correlation between the number of exposure cycles to 405 nm light and the susceptibility to photoinactivation *in vitro* for any of the three gram-negative bacteria examined, *P. aeruginosa*, *A. baumannii*, or *E. coli* (16).

The effect of the blue light on drug-resistant strains of bacteria is also of significant consequence, as the growing issue of antibiotic-resistant bacteria presents a need for novel methods of controlling bacterial spread (18). Multiple studies have shown the blue light to have efficacy in inactivating multidrug-resistant bacteria (18–22). In a 2010 study that administered high-intensity narrow-spectrum light (HINS-light) with a peak wavelength of 405 nm on a hospital isolation room with high levels of *S. aureus* and methicillin-resistant *S. aureus* (MRSA), the use of HINS-light was associated with a reduction from 0.84 colony forming units/square centimeter (CFU/cm^2^) to 0.42 CFU/cm^2^ in *S. aureus* count, and a reduction from 0.73 to 0.26 CFU/cm^2^ in MRSA count (22). Barneck et al. found 405-nm light to be effective in inactivating *E. coli* that had been conferred with ampicillin resistance, with an 82% mean reduction after 120 min of exposure at an irradiance of 2.89 mW/cm^2^, and a 100% mean reduction after 120 min of exposure at an irradiance of 9.37 mW/cm^2^ (19). Bumah et al. observed antimicrobial blue light of wavelengths 405 and 470 nm to be effective in reducing MRSA (20). A trend seen in this study was that greater fluences of light were required to achieve 100% reduction in the more dense (greater CFU/mL) bacterial colonies (20). In an *in vitro* study, suspensions of multidrug-resistant strains of *E. coli* were seen to be susceptible to photoinactivation by 410 nm light, with all strains exhibiting a greater than 3-log_10_ reduction after 180 min of the light exposure (21). The strains of *E. coli* used in this study were resistant to colistin, broad spectrum cephalosporin, and carbapenem antibiotics that are considered the last resort for controlling multidrug-resistant bacteria (21). Leanse et al. proposed that antimicrobial blue light could be used in conjunction with quinine hydrochloride to inactivate multi-drug resistant bacteria (18). The use of quinine hydrochloride in conjunction with 405 nm light irradiation was associated with a greater than 10^3^-fold reduction in planktonic suspensions of multidrug-resistant strains of *P. aeruginosa* and *A. baumanii* (18). It has also been proposed that sublethal doses of visible blue light can be used in conjunction with traditional antibiotics to treat multidrug-resistant bacteria (23). The findings of Wozniak et al. showed extensively drug-resistant *A. baumannii* to have enhanced susceptibility to antibiotics after sublethal exposure to the antimicrobial blue light (23).

There is consensus among studies that there is a difference in susceptibility to 405-nm light between gram-positive and gram-negative bacteria, with gram-positive bacteria typically being the more vulnerable and gram-negative bacteria requiring relatively greater doses of 405-nm light for inactivation (11, 24). McKenzie et al. study found that *L. monocytogenes*, a gram-positive bacterium, had greater susceptibility to inactivation by 405 nm light compared to *E. coli*, a gram-negative bacterium (11). After culture in optimal growth conditions, complete inactivation of 5-log_10_ populations was achieved with a dose of 84 J/cm^2^ 405 nm light for *L. monocytogenes*, whereas a greater dose of 378 J/cm^2^ was required to achieve the same extent of inactivation of the *E. coli* populations (11). A contrasting finding was observed in a 2016 study which examined the relative bactericidal effects of 405 nm light against four different clinically relevant bacteria and found gram-positive and gram-negative bacteria to have similar susceptibility to 405 nm light, with no statistically significant differences in susceptibility between the species tested (19). This study found ampicillin-resistant *E. coli* to be the most susceptible to photoinactivation by 405 nm light, followed by *S. pneumoniae*, *S. aureus*, and *P. aeruginosa* (19). This departure from the more widely observed tendency of gram-positive bacteria to have greater susceptibility to 405 nm light than gram-negative bacteria was attributed to possible variability in species, strains, and stain family (19). Since Gram-positive bacteria have a thick peptidoglycan layer and no outer lipid membrane whilst Gram-negative bacteria have a thin peptidoglycan layer and have an outer lipid membrane, the observed differential responses of the bacteria to the blue light could potentially be attributable to the differences between the cell membranes of Gram-positive and Gram-negative bacteria. Further investigations in this area are warranted.

## Fungal inactivation potential of the blue light

The blue light has demonstrated efficacy across several studies in inactivating fungal pathogens. Studies have shown the blue light to be effective in inactivating *Candida albicans*, a clinically relevant fungal pathogen responsible for commonly seen fungal infections (2, 25). It has been proposed that endogenous photosensitizers may be responsible for the phototoxicity effect of antimicrobial blue light on *C. albicans*, as fluorescence spectroscopy has indicated the presence of porphyrins and flavins in *C. albicans* (2). One such study administered 405 nm light to *ex vivo* rabbit corneas with *C. albicans* keratitis and concluded that it was indeed an effective antifungal treatment and a viable option for treatment pending studies investigating its safety for the surrounding eye tissue (25). *In vitro* experiments conducted by Zhang et al. exposing both human keratinocytes and *C. albicans* to 415 nm light showed that *C. albicans* was more susceptible than human keratinocytes to inactivation upon light exposure, and that there is a therapeutic window of exposure wherein *C. albicans* cells will be inactivated and human keratinocytes unharmed (2). In the *in vivo* experiment by Zhang et al., administering 415 nm light to *C. albicans*-infected mouse burn wounds, the light exposure substantially reduced the fungal burden, although there was a recurrence of infection following the cessation of light treatment (2).

In a study using *Saccharomyces cerevisiae* as the model organism, it was found that a one log_10_ reduction of the pathogen could be achieved with a 182 J/cm^2^ dose of 405 nm light, and with a 526 J/cm^2^ dose of 450 nm light (26). Subsequent analysis of trypan blue staining produced no evidence of damage to the cell membranes of the light-treated samples, suggesting that the visible blue light treatment did not impair the viability of the fungal cells but rather impacted their ability to be cultured (26). Analysis of the *S. cerevisiae* cell lysates indicated that the presence of the endogenous photosensitizer protoporphyrin IX may play a role in the sensitivity of the fungal cells to 405 nm light (26). The sensitivity of the fungal cells to 450 nm could conceivably be explained by the presence of riboflavin and other flavins (26).

An area for future investigation within the application of antimicrobial blue light to fungal pathogens is the impact of melanization (2). Fungal pathogens like *C. albicans* have been known to produce melanin in the context of an infection, which could conceivably shield fungal cells from light exposure and thus impact the efficacy of fungal inactivation by antimicrobial blue light (2). Future studies investigating the interaction between this phenomenon and antimicrobial blue light will be necessary to more fully ascertain whether antimicrobial blue light is a viable disinfecting strategy for fungal pathogens in public health and clinical applications.

## Viral inactivation potential of the blue light

Because virions are known not to contain endogenous porphyrins, there is relatively less understanding of the mechanism and efficacy of antimicrobial blue light in inactivation of viruses compared to bacteria and fungi (27). There have been studies investigating the potential of the blue light for inactivating human norovirus, which is a leading cause of acute gastroenteritis and can be transmitted in a variety of ways including contaminated food and surfaces (27, 28). A major limitation of studies that seek to investigate the inactivation of norovirus, an issue of substantial clinical and public health importance, is that there is currently no standardized protocol for propagation of human norovirus, and experimental studies must be conducted using a surrogate virus (27, 28). Recently for the first time, noroviruses were shown to replicate in stem-cell derived human enteroids that would pave the way for a more systematic *ex vivo* culture of this group of viruses in the long-term (29).

A 2017 study sought to investigate the potential of the blue light as a preventative tool for norovirus outbreak in hospital settings, using Feline Calicivirus (FCV) as a surrogate for human norovirus (27). Female embryonic cells were inoculated with FCV and incubated, and after 90% of the cell monolayer was destroyed, the virus-containing supernatant was collected and stored (27). The media in which the virus-containing supernatant was suspended during exposure to the 405 nm light treatment appeared to affect the efficiency and extent of virus inactivation, with minimal media requiring the greatest dose, presumably because of the absence of porphyrins both in the virus and in the medium (27).

It was seen that FCV suspended in organically rich media or biologically relevant organically rich media showed the greatest reductions in FCV and necessitated relatively lower doses of 405-nm light (27). These findings support the basic principle that the presence of porphyrins enhances inactivation by 405 nm light, as the organically rich and biologically relevant media contained porphyrins, whereas minimal media did not contain porphyrins and showed relatively lower efficiency in FCV inactivation (27). The greater susceptibility of FCV when in the presence of biologically relevant substances including plasma and artificial saliva suggest that 405 nm light could potentially be a promising microbicidal tool in the contexts, where virus contaminants are likely to be in the presence of biological media containing endogenous photosensitizers.

Another study which used Tulane virus as a surrogate for human norovirus in the context of food safety and the decontamination of the produce also found that viral inactivation by 405 nm light was limited in the absence of some form of enhancer (28). Although the singlet oxygen enhancers riboflavin and rose Bengal did appear to enhance viral inactivation by 405 nm light, the overall extent of inactivation was limited, and there is a need for further research into food-grade compounds that can more substantially enhance the production of reactive oxygen species and subsequently inactivate viruses (28) while the basic mechanism of 405 nm light inactivation of microbes is implicated through the production of radical oxygen species by the excitation of porphyrins or flavins present either within the organism or in the surrounding environment as suggested in section Mechanism of pathogen inactivation by the blue light of this review.

## The blue light potential in inactivation of biofilms including drug-resistant biofilms

Biofilms are aggregations of microorganisms that are understood to be an adaptive mechanism to improve survival under conditions of stress (29). Biofilms have been observed to form on both living and non-living surfaces in clinical settings, and their increased resistance to antimicrobial treatments and host immune response present a considerable risk for the spread of infectious diseases (29, 30). The increased resistance to stress conditions observed in bacterial biofilms is thought to be attributable to the formation of an extracellular polymeric substance (EPS) that envelops bacteria (29, 31, 32). Polymicrobial biofilms, biofilms composed of more than one species of microorganism, are thought to have increased antimicrobial resistance attributable to the transfer of antimicrobial resistance genes between microorganisms (33). Studies have investigated the use of the blue light as an microbicidal strategy targeting both monomicrobial and polymicrobial biofilms.

The blue light wavelengths have been observed to inactivate bacterial biofilms, as seen in a 2020 study which concluded that 400 nm light effectively inactivated *Pseudomonas fluorescens* and *S. epidermidis* biofilms (34). Ferrer-Espada et al. concluded that 405 nm light exhibits efficacy in inactivating monomicrobial biofilms, with the *A. baumannii*, *S. aureus*, *N. gonorrhoeae*, and *P. aeruginosa* biofilms being the most susceptible to inactivation (31). It has also been shown that the monomicrobial biofilms composed of multidrug-resistant strains of *A. baumanni* and *P. aeruginosa* could also be inactivated with the 405 nm light treatment (31). In a study that applied 405-nm visible blue light to monomicrobial and polymicrobial biofilms, significant bacterial reductions were observed after light treatment (33). Monomicrobial biofilms of *P. aeruginosa*, *C. albicans*, and methicillin-resistant *S. aureus* were subjected to 405 nm light at a dose of 500 J/cm^2^, and the resultant inactivation in CFU were 6.30log_10_, 2.33log_10_, and 3.48log_10_, respectively (33). The polymicrobial biofilms composed of *P. aeruginosa* and *C. albicans* exhibited a 6.34log_10_ inactivation in *P. aeruginosa* and a 3.11log_10_ CFU inactivation in *C. albicans* after exposure to the same 500 J/cm^2^ dose of 405 nm light (33). In the polymicrobial biofilms composed of *P. aeruginosa* and methicillin-resistant *S. aureus*, the same 500 J/cm^2^ dose of 405 nm light led to a 3.40log_10_ CFU inactivation of *P. aeruginosa* and a 2.37log_10_ CFU inactivation of methicillin-resistant *S. aureus* (33). This study demonstrated the efficacy of 405 nm blue light against polymicrobial biofilms and suggests the importance of future *in vivo* studies to determine whether this could be applied to the clinical setting in the context of wound healing (33).

A study by Liu et al. applied 405 nm light to biofilms of *Moraxella catarrhalis*, a common causative pathogen for otitis media seen in children which is also known to form biofilms in the middle ear (32). Upon exposure to 405 nm light at a dose of 216 J/cm^2^, a reduction in biofilms of greater than 3log_10_ CFU was observed (32). Scanning electron microscopy analysis of the light-treated *M. catarrhalis* biofilms showed evidence of damage to the pathogen cell walls and loss of the EPS which provides structure to the biofilms (32). The findings of this study suggest a potential for 405 nm light to be used as a treatment for *M. catarrhalis* associated otitis media, pending further investigation to determine a suitable mode of delivery of 405 nm light to the middle ear in the clinical setting (32).

A 2020 study investigated the effect of visible blue light on *Pseudomonas aeruginosa* biofilms, which pose a significant risk of nosocomial infection in the clinical environment (35). It was observed that 410 nm light at a low dose of 75 J/cm^2^ effectively prevented the formation of the biofilm matrix structure, and the higher doses of 410 nm light at 225 and 450 J/cm^2^ were observed to cause damage to the bacterial cells themselves and impair further cell growth (35). The elimination of existing biofilms by 410 nm light was observed to be dose-dependent, with greater doses of light being associated with greater efficacy in decreasing the biofilm matrix, detaching the bacterial cells, and inactivating the bacterial cells (35).

## The blue light potential in food safety from pathogens

Foodborne illness is a major public health concern in the United States, and proper prevention of these illnesses necessitate the use of effective disinfection techniques to ensure the safety of the food supply (24). Existing decontamination methods including thermal control, ultraviolet light, and chemical sanitizers have raised concerns about reduced nutritional value and carcinogenicity of the food product (24). Ultraviolet light has been demonstrated to be efficacious for decontamination in the food industry in contexts such as juice pasteurization and meat treatment, although its viability for widespread use in the food industry is limited by factors including safety concerns, limited ability to penetrate through packaging material, and potential for degradation of plastics (11, 36). A 2016 study examined the viability of the blue light treatment as a food disinfection technique by administering different doses of the light to processed meat inoculated with *E. coli* and to cucumbers inoculated with *Salmonella* Typhimurium (24). In both treatment conditions, there was an observed increase in inactivation rate of the bacteria with increasing fluence, with complete inactivation of the *S.* Typhimurium on cucumber peels with administration of 464 nm light at 18 J/cm^2^, and 96.3% inactivation of *E. coli* on processed meat with administration of 405 nm light at 100 J/cm^2^ (24). While this study establishes that antimicrobial blue light is effective against *E. coli* and *S.* Typhimurium present on the food surfaces, it remains to be seen whether the blue light has any side effects on the quality and safety of the light-treated food items themselves (24).

A McKenzie et al. study subjected *E. coli* and *L. monocytogenes* to sub-lethal stress conditions that mimic the conditions that bacterial contaminants would likely be exposed to in the food services environment, including hot and cold temperatures, acidic environments, and high salt concentration environments (11). This study found that with both *E. coli* and *L. monocytogenes*, bacterial populations that experienced sublethal damage were more susceptible to inactivation by 405 nm light compared to bacterial populations grown in optimal conditions (11). Investigators propose that the observed enhancement in the bactericidal effects of 405 nm light when applied in conjunction with sub-lethal stress suggests a potential application of 405 nm light for decontamination of the food processing environment and reduction of the spread of foodborne pathogens (11).

Human norovirus is another major cause of foodborne illness, and a 2017 study sought to evaluate 405 nm light as a potential preventative tool by examining its effects on Tulane virus (a human norovirus surrogate) present on the surface of blueberries (28). Because viruses do not contain porphyrin, a major photosensitizer involved in the inactivation of bacteria and fungi by antimicrobial blue light, investigators in this study added food-grade singlet oxygen enhancers to the blueberries to enhance the production of the reactive oxygen species and thereby enhance the inactivating effect of the 405 nm light (28). Blueberries were inoculated with the Tulane virus, treated with singlet oxygen enhancers, and subjected to 30 min of 405 nm light exposure (28). In blueberries prepared with the singlet oxygen enhancer riboflavin, the 405 nm light treatment group exhibited greater inactivation of Tulane virus, with a statistically significant difference between the treated and untreated group (28). Among blueberries prepared with the singlet oxygen enhancer rose Bengal, the 405 nm light-treated group showed greater inactivation of the virus, although the difference between the treated and untreated group was not statistically significant (28). Investigators concluded that there is a need for further research of singlet oxygen enhancers that can be used in conjunction with 405-nm light to inactivate a virus more substantially like human norovirus and help ensure the decontamination of produce (28).

## The blue light potential in decontaminating surfaces and environment

Nosocomial infections, also referred to as healthcare-associated infections (HAI) are a cause for increased morbidity and mortality of hospital patients, and their prevention necessitates safe and effective methods of environmental decontamination within the hospital (37, 38). Nosocomial infections are also associated with prolonged hospital stay and increased healthcare costs, and new technologies for the decontamination of surfaces and equipment could potentially help to reduce the burden of disease attributable to nosocomial infection (38). The blue light has been investigated as a tool for environmental decontamination, as exemplified by the high-intensity narrow-spectrum light environmental decontamination system (HINS-light EDS) which uses a peak wavelength of 405 nm and has been shown across studies to have efficacy with inactivating environmental pathogens (22, 37, 39).

Burns patients are particularly susceptible to infection due to the immunosuppressed state and impaired integrity of the skin, and the complications can include sepsis and organ failure (12, 37, 40). Burns patients are also likely to disperse bacteria into the surrounding environment, which makes infection control practices in burns units vitally important (12, 37). It has been observed that infection control practices in burn units have helped reduce fatality rates of burns patients (41). Maclean et al. examined the efficacy of 405 nm light for disinfecting a hospital isolation room in the hospital burns unit (22). Two such light sources were fitted in the ceiling of the hospital isolation room, and bacterial growth was examined in an unoccupied room, in an occupied room with intermittent 405 nm light application, in an occupied room with constant 405 nm light application, and in an occupied room without 405 nm light (22). Bacteria were sampled from frequently touched surfaces in the isolation room and examined after a 48-h incubation period (22). Results showed that the 405 nm light effected a statistically significant reduction in bacterial counts in each experimental condition (22). It was also noted that bacterial counts remained low for a period after discontinuation of the light application in the unoccupied room, in contrast to the occupied room which exhibited an increase in bacterial contamination up to pre-treatment levels within 2 days of the discontinuation of 405 nm light treatment (22). As this study administered the 405 nm light treatment concurrently with existing hospital decontamination practices, it was demonstrated that the 405-nm light treatment successfully achieved substantial bacterial decontamination that exceeds those achieved by currently used hospital decontamination practices alone, and thus provides strong evidence in support of the application of 405-nm light as a supplementary hospital decontamination method in the setting of a burn unit hospital environment (22).

Another study applied 405 nm light EDS to an inpatient burn isolation room occupied by a single burns patient and to an outpatient burn clinic room with 7–12 burns patients (37). As with the 2010 Maclean et al. study, samples were collected from several frequently touched surfaces and examined after a 48-h incubation period (37). With 14-h treatment of HINS-light for two consecutive days, a 27–75% reduction in bacterial CFU was observed from samples collected at 08:00 h from the inpatient burn isolation rooms (37). In the study conducted on the outpatient burn clinic rooms in which multiple patients were treated for burn wounds, the 8-h application of 405 nm light was associated with a lesser increase in bacterial contamination after the clinic closed; the outpatient room without 405 nm light exhibited a mean increase of 14.1 CFU/plate during clinic operation, whereas the outpatient room with 8-h 405 nm light treatment exhibited a mean increase of only 5.5 CFU/plate during clinic (37). Although complete bacterial inactivation was not achieved, this study provides further evidence of the efficacy of violet blue light in its potential application as a supplementary environmental decontamination tool in the inpatient and outpatient burn treatment settings to help prevent further spread of nosocomial infections (37). The results of the outpatient clinic experiment indicated that the 405-nm light treatment may be a promising decontamination tool for communal environments where cross-contamination by multiple individuals may be a concern (37). Bache et al. also applied 405 nm light treatment to a hospital burns unit at different irradiance levels and for different durations of exposure (12). While no correlation was observed between irradiance level and bacterial reduction, it was observed that increased duration of exposure was correlated with increased reduction in bacteria (12). As in the 2010 study by Maclean et al. and the 2011 study by Bache et al. the 2017 study demonstrated a decrease in bacterial counts upon exposure to 405 nm light treatment, and an increase in bacterial counts following the discontinuation of 405 nm light treatment (12).

Maclean et al. tested the 405-nm light technology in an intensive care unit (ICU) (39). An ICU room occupied by a patient for 9 days prior to the start of the study exhibited a 67% mean percentage reduction in *staphylococcal* counts following 405 nm light treatment (39). A second experiment with a patient who had arrived at the ICU room just 12 h prior to the start of the study did not exhibit a statistically significant mean percentage reduction in *staphylococcal* counts after 405 nm light exposure, and this was thought to be due to a lower starting level of bacterial contamination as the room had been cleaned more recently than in the previous experiment (39). Samples collected 24 h following the discontinuation of 405 nm light exposure exhibited a 357% increase in *staphylococcal* count, which supports the hypothesis that the 405 nm light has a substantial decontaminating effect (39). In a third experiment, the effect of direct vs. indirect exposure to the 405 nm light was examined (39). The sampled surfaces under direct exposure were observed to have a 63% mean reduction in bacterial counts associated with HINS-light treatment, and a 94% mean increase after discontinuation of the 405 nm light (39). The sampled surfaces under indirect exposure to 405 nm light were observed to have a 48% mean reduction in bacterial counts, and a 71% mean increase after discontinuation of 405 nm light (39). These results showed that both direct and indirect exposure to 405 nm light is associated with a significant reduction in bacterial contamination, although direct exposure does lead to a greater extent of bacterial inactivation (39).

A 2017 study applied visible blue light to the issue of indoor air pollution, and specifically aimed to degrade gaseous formaldehyde which can be injurious to health (42). Investigators first synthesized a titanium dioxide photocatalyst doped with the elements W, Ag, N, and F such that it would have photocatalytic activity in the visible light wavelengths, then combined the photocatalyst with formaldehyde in test tube reactors which were then exposed to visible blue light (42). Under this experimental setup, it was observed that the combined multi-element doped titanium dioxide was the most effective catalyst with 88.1% photocatalytic degradation of gaseous formaldehyde (42). The findings of this study suggest a potentially promising application of visible blue light in conjunction with photocatalysts for the purification of air (42).

## The blue light potential in clinical applications to control localized infections without harm to human cells

Multiple studies examining the viability of the blue light for pathogen inactivation in clinical contexts have observed that photoinactivation of clinically relevant pathogens can be achieved at lower dosages of the blue light relative to the dosages required to cause damage to human cells (2, 3, 6, 32). A couple of studies by Wang et al. observed a selectivity of 405-nm blue light for *N. gonorrhoeae* cells over human vaginal epithelial cells, which supports its potential application as a treatment for *gonococcal* infections (3, 6). These studies include analysis of fluorescence emission spectra and ultra-performance liquid chromatography of normal human vaginal epithelial cells (VK2/E6E7) after exposure to 405 nm blue light (3). The co-cultures of *N. gonorrhoeae* cells and vaginal epithelial cells exposed to 108 J/cm^2^ 405 nm light showed a substantial increase in non-viable *N. gonorrhoeae* cells (from 32.85 ± 7.70% before light exposure to 78.15 ± 6.52% after light exposure), with no notable increase in the percentage of non-viable vaginal epithelial cells (3). Fluorescence emission spectra showed a small peak at 460 nm, indicating the presence of flavins, and otherwise minimal absorbance between 350 and 700-nm, indicating minimal presence of porphyrins (3). The ultra-performance liquid chromatography analysis showed that VK2/E6E7 vaginal epithelial cells had substantially lower concentrations of endogenous flavins FAD, FMN, and riboflavin compared to ATCC 700825 *N. gonorrhoeae* cells with total flavin concentrations of 28.12 and 363.63 nmol/g respectively (3). Thus, it appears that the selective inactivation of *N. gonorrhoeae* cells over normal human vaginal epithelial cells upon exposure to 405 nm light can be explained by the increased presence of endogenous photosensitizers in the bacterial cells (3). Investigators proposed administration of 405 nm light via a laser with an optical fiber diffuser directed to the vaginal canal, similar to the present mode of administration of photodynamic therapy for treatment of condyloma acuminata caused by a human papilloma virus (HPV) in the vagina (3); further investigations by the same authors concluded that although infected vaginal epithelial cells were more vulnerable to 405 nm light than non-infected vaginal epithelial cells, the greater susceptibility of *N. gonorrhoeae* cells to 405 nm light indicated a possible therapeutic window of 405 nm light exposure whereby *N. gonorrhoeae* would be selectively inactivated, and the infected vaginal epithelial cells would be largely unharmed (6).

In a 2016 study investigating the potential of 415 nm light as a treatment for *C. albicans* keratitis, *C. albicans* was observed to be substantially more sensitive to photoinactivation than human keratinocytes, further reinforcing the idea of a therapeutic window in which the blue light can eliminate pathogens without harming normal human cells (2). Ferrer-Espada et al. and Liu et al. both observed that at an exposure dose of 216 J/cm^2^ of the blue light, there was no significant reduction in the viability of human keratinocytes (32, 33), whereas the same dosage of light caused significant reduction of viability in the polymicrobial biofilms (33). A greater dosage of 405 nm light, 500 J/cm^2^ was observed to cause cytotoxicity in the human keratinocyte cells (33). Another study using 415 nm blue light also observed multidrug-resistant *A. baumannii* to be substantially more susceptible to photoinactivation compared to human keratinocytes (17).

## The blue light potential in prevention and treatment of eye infections

Recent studies have explored the potential application of the blue light for prevention and treatment of infection relating to the eyes (25, 43–45). Zhu et al. tested the efficacy of 415 nm light for the treatment of keratitis in an *ex vivo* rabbit model and an *in vivo* mouse model, where the causative agent was a bioluminescent strain of *P. aeruginosa* (43). In the experiment with *ex vivo* rabbit corneas, it was seen that the blue light treatment effected the greatest extent of reduction of bacterial luminescence when administered 6 h after inoculation, compared to 24 h after inoculation (43). There was no recurrence of infection observed in the rabbit corneas after antimicrobial light treatment (43). A similar *ex vivo* study of keratitis induced in rabbit corneas with a bioluminescent strain of *Candida albicans* found that 405 nm light was effective in substantially reducing the fungal infection (25). In the *in vivo* mouse study, complete bacterial luminescence eradication was achieved with treatment both 6 and 24 h after inoculation, although recurrence of infection was observed in both groups with the 24-h post inoculation treatment group exhibiting the more severe recurrence of infection (43). The observation that infection recurred in the *in vivo* experiment with mice and not in the *ex vivo* experiment with rabbit corneas suggests that the eyelids of mice in the *in vivo* experiments may have covered parts of the cornea during the 415 nm light exposure, thus preventing all the bacteria from being exposed to the light (43). The findings from this study provide evidence of the potential of 415 nm light as a treatment for *P. aeruginosa* keratitis, although further studies are needed to establish sound methods of adequately irradiating all the cornea in the *in vivo* context and mitigating the recurrence of infection after light treatment.

## Discussion

This review has presented an overview of the existing understanding of visible blue light about its mechanism of microbicidal action, efficacy with inactivating different pathogens and degrading contaminants, as a potential multipurpose microbicidal tool for applications in clinical medicine and public health contexts, while providing safety for host cells or human exposure, unlike ultraviolet light (UV)-based technologies that also damage host environment. This study has also provided an assessment of the viability of potentially implementing the blue light as a disinfecting protocol in settings such as environmental decontamination, food safety, and treatment of localized infections.

Bacteria are the most thoroughly investigated and well-understood pathogen as a target of the blue light for bactericidal purposes. A widely accepted theory of the mechanism of inactivation of bacteria is that the blue light causes the photoexcitation of endogenous photosensitizers, namely porphyrins and flavins, which then precipitate the release of reactive oxygen species leading to toxicity and cell death (1, 3). There also appears to be consensus in the field that the sensitivity of a bacterium to a given wavelength of visible blue light may be affected by the type and prevalence of endogenous photosensitizers within the bacterium, and the wavelength of peak absorbance specific to the endogenous photosensitizers that are present (1, 3). Porphyrins have been noted to have peak absorbance around a wavelength of 405-nm, and flavins around 470 nm, and the relative abundance of these two endogenous photosensitizers may contribute to the sensitivity of the bacterium to photoinactivation by certain wavelengths of visible blue light (1, 3). While porphyrins and flavins seem to be the most ubiquitous photosensitizers, there has also been evidence of the possible involvement of other photosensitizers in the photoinactivation of bacteria by antimicrobial blue light (1). An observed characteristic of bacterial inactivation by 405 nm antimicrobial blue light across studies is the greater susceptibility of gram-positive bacteria compared to gram-negative bacteria, with gram-negative bacteria generally requiring greater doses of light for inactivation (24). Antimicrobial blue light has also shown promise for addressing the issue of bacterial resistance, as studies using *N. gonorrhoeae* and *S. aureus* have shown no significant change in bacterial reduction in response to 405 nm light even after repetitive exposure to successive cycles of high-intensity light (5, 6). The finding that *S. aureus* exposed to 405 nm light at a low-level during culture exhibited a level of tolerance to inactivation by high-intensity 405 nm light suggests that bactericidal levels of light should be used in practice and indicates a need for further investigation of the mechanism through which this tolerance is developed (5).

Studies with fungi have shown antimicrobial blue light to have significant efficacy with reducing fungal burden (2, 25, 26). The presence of endogenous photosensitizers including porphyrins and flavins in *C. albicans* has been observed with fluorescence spectroscopy, which suggests the possibility that these photosensitizers play a role in the photoinactivation of *C. albicans*, as has been theorized to be the mechanism of photoinactivation in bacteria (2). Studies using antimicrobial blue light of wavelength 405 and 415 nm have observed *C. albicans’* susceptibility at both wavelengths (2, 25). There is a need for further investigation of fungal inactivation by antimicrobial blue light to characterize the effects of melanization by fungi in the context of infection, as this could potentially limit the efficacy of antimicrobial blue light (2). While there is evidence of fungal susceptibility to antimicrobial blue light, further investigation will be necessary to determine whether antimicrobial blue light is a viable antifungal treatment in practice.

Studies of viral inactivation by antimicrobial blue light also show some degree of antiviral efficacy. Because virions, unlike bacteria and fungi, do not contain endogenous porphyrins, there is relatively more ambiguity surrounding the mechanism and efficacy of photoinactivation (28). It has been observed that photoinactivation of viruses by antimicrobial blue light in the absence of other factors is limited but is enhanced in the presence of singlet oxygen enhancers or nutrient-rich media containing photosensitive compounds (27, 28).

The blue light has also been investigated as a tool for eliminating biofilms, which are of significant concern in contexts including environmental decontamination and treatment of localized infections due to their characteristic durability and decreased sensitivity to conventional antimicrobials (29, 30, 32). Studies show that treatment with visible blue light results in significant reduction in bacterial CFU when applied to monomicrobial and polymicrobial biofilms (32, 33, 35).

Several studies have also shown that visible blue light could be a promising tool for environmental decontamination and non-clinical applications. Studies in food safety have shown visible blue light to be effective in reducing the bacterial or viral burden on the surface of food products, although there is a need for more research to optimize the antimicrobial effects and understand potential effects on the quality of the food products themselves (24, 28). The blue light has been shown in multiple studies to be effective for reducing the bacterial burden within the clinical environment, as seen in studies of hospital isolation rooms, inpatient and outpatient burn treatment settings, and the hospital intensive care unit (12, 22, 37, 39). There appears to be agreement amongst studies that visible blue light is a viable tool that could potentially supplement existing hospital decontamination practices and help reduce the spread of nosocomial infections (12, 22, 37, 39). In addition, visible blue light has been shown to be effective when used in conjunction with a novel photocatalyst to degrade gaseous formaldehyde, a major contributor to indoor air pollution that is injurious to health (42).

Numerous studies have assessed the potential of the blue light for use in clinical applications, including treatment of gonococcal infections, localized infections, and wound healing. A common finding among these studies is that pathogens appear to have greater susceptibility to visible blue light than normal host cells, such that there is a therapeutic window of exposure within which the pathogen would be selectively inactivated, and the host cells preserved (2, 3, 6). These findings suggest that the blue light could indeed be a viable treatment for infection in the clinical setting. The observation of recurrence of infection after light treatment seen in more than one *in vivo* study is of concern and suggests that further investigation is needed to quantitate the prevalence of pathogen more effectively in the target area and more effectively administer the light to the entirety of the infected area (2, 43).

The existing literature supports the efficacy of visible blue light as an microbicidal tool in several applications including environmental decontamination and treatment of clinically relevant pathogens. It has been widely observed that although human cells are vulnerable to visible blue light at high doses, the doses that are lethal to many clinically relevant pathogens do not appear to cause damage to human cells (2, 3, 6). Although further studies will be needed to determine with more certainty the impact on normal human tissue and disease progression, current evidence points toward the viability of visible blue light for treatment of infections including otitis media, keratitis, gonococcal infections, and wound infections. Multiple studies have observed the blue light to have efficacy in inactivating multidrug-resistant bacteria, suggesting that the use of this light can potentially help to slow the spread of antibiotic resistance and untreatable infections (18, 20, 21, 23). Further research is necessary to adequately understand the mechanism of inactivation for fungal and viral pathogens to better inform the way the blue light can be optimized to eliminate these pathogens in contexts such as the localized treatment of infections and decontamination of food and food processing environments. Regarding the application of the blue light to disinfection of produce and other food products, further investigation will be needed to determine any adverse impacts on the nutritional content and shelf life of the light-treated foods. Another direction for future research in the use of the blue light as an microbicidal tool is to further identify new factors that can be used in conjunction with the blue light to potentiate and enhance its microbicidal effects.

## Author contributions

DH received guidance and mentoring by CA in writing the manuscript. CA reviewed and edited the manuscript for publication. Both authors approved the submitted version.

## Conflict of interest

The authors declare that the research was conducted in the absence of any commercial or financial relationships that could be construed as a potential conflict of interest.

## Publisher’s note

All claims expressed in this article are solely those of the authors and do not necessarily represent those of their affiliated organizations, or those of the publisher, the editors and the reviewers. Any product that may be evaluated in this article, or claim that may be made by its manufacturer, is not guaranteed or endorsed by the publisher.

## References

[1] Hessling M Wenzel U Meurle T Spellerberg B Hönes K. Photoinactivation results of *Enterococcus moraviensis* with blue and violet light suggest the involvement of an unconsidered photosensitizer. *Biochem Biophys Res Commun.* (2020) 533:813–7. 10.1016/j.bbrc.2020.09.091 32993958

[2] Zhang Y Zhu Y Chen J Wang Y Sherwood ME Murray CK Antimicrobial blue light inactivation of *Candida albicans*: in vitro and in vivo studies. *Virulence.* (2016) 7:536–45. 26909654 10.1080/21505594.2016.1155015 PMC5026794

[3] Wang Y Ferrer-Espada R Baglo Y Gu Y Dai T. Antimicrobial blue light inactivation of *Neisseria Gonorrhoeae*: roles of wavelength, endogenous photosensitizer, oxygen, and reactive oxygen species. *Lasers Surg Med.* (2019) 51:815–23. 10.1002/lsm.23104 31157931 PMC6922002

[4] Amin RM Bhayana B Hamblin MR Dai T. Antimicrobial blue light inactivation of *Pseudomonas Aeruginosa* by photo-excitation of endogenous porphyrins: in vitro and in vivo studies. *Lasers Surg Med.* (2016) 48:562–8. 10.1002/lsm.22474 26891084 PMC4914480

[5] Tomb RM Maclean M Coia JE MacGregor SJ Anderson JG. Assessment of the potential for resistance to antimicrobial violet-blue light in *Staphylococcus aureus*. *Antimicrob Resist Infect Control.* (2017) 6:100. 10.1186/s13756-017-0261-5 29046782 PMC5639585

[6] Wang Y Ferrer-Espada R Baglo Y Goh XS Held KD Grad YH Photoinactivation of *Neisseria Gonorrhoeae*: a paradigm-changing approach for combating antibiotic-resistant gonococcal infection. *J Infect Dis.* (2019) 220:873–81. 10.1093/infdis/jiz018 30629196 PMC6667797

[7] Scallan E Hoekstra RM Angulo FJ Tauxe RV Widdowson M Roy SL Foodborne illness acquired in the United States—major pathogens. *Emerg Infect Dis.* (2011) 17:7–15. 10.3201/eid1701.p11101 21192848 PMC3375761

[8] Otter JA Vickery K Walker JT DeLancey Pulcini E Stoodley P Goldenberg SD Surface-attached cells, biofilms and biocide susceptibility: implications for hospital cleaning and disinfection. *J Hosp Infect.* (2014) 89:16–27. 10.1016/j.jhin.2014.09.008 25447198

[9] Ramakrishnan P Maclean M MacGregor SJ Anderson JG Grant MH. Cytotoxic responses to 405nm light exposure in mammalian and bacterial cells: involvement of reactive oxygen species. *Toxicol Vitro.* (2016) 33:54–62. 10.1016/j.tiv.2016.02.011 26916085

[10] Endarko E Maclean M Timoshkin IV MacGregor SJ Anderson JG. High-intensity 405nm light inactivation of *Listeria monocytogenes*. *Photochem Photobiol.* (2012) 88:1280–6. 22582879 10.1111/j.1751-1097.2012.01173.x

[11] Mckenzie K Maclean M Timoshkin IV Macgregor SJ Anderson JG. Enhanced inactivation of *Escherichia Coli* and *Listeria monocytogenes* by exposure to 405 Nm light under sub-lethal temperature, salt and acid stress conditions. *Int J Food Microbiol.* (2014) 170:91–8. 24291187 10.1016/j.ijfoodmicro.2013.10.016

[12] Bache SE Maclean M Gettinby G Anderson JG MacGregor SJ Taggart I. Universal decontamination of hospital surfaces in an occupied inpatient room with a continuous 405 nm light source. *J Hosp Infect.* (2018) 98:67–73. 10.1016/j.jhin.2017.07.010 28716671

[13] Institute of Medicine (Us) Forum on Microbial Threats. *Antibiotic Resistance: Implications for Global Health and Novel Intervention Strategies: Workshop Summary.* Washington, DC: National Academies Press (2010). 21595116

[14] Wang Y Harrington OD Wang Y Murray CK Hamblin MR Dai T. In vivo investigation of antimicrobial blue light therapy for multidrug-resistant *Acinetobacter Baumannii* burn infections using bioluminescence imaging. *J Vis Exp.* (2017) 22:54997. 10.3791/54997 28518072 PMC5523652

[15] Fauci AS Morens DM Folkers GK. The challenge of emerging and re-emerging infectious diseases. *Nature.* (2010) 430:242–9. 10.1038/nature02759 PMC7094993 15241422

[16] Leanse LG Harrington OD Fang Y Ahmed I Goh XS Dai T. Evaluating the potential for resistance development to antimicrobial blue light (at 405 Nm) in gram-negative bacteria: in vitro and in vivo studies. *Front Microbiol.* (2018) 9:2403. 10.3389/fmicb.2018.02403 30459719 PMC6232756

[17] Zhang Y Zhu Y Gupta A Huang Y Murray CK Vrahas MS Antimicrobial blue light therapy for multidrug-resistant *Acinetobacter Baumannii* infection in a mouse burn model: implications for prophylaxis and treatment of combat-related wound infections. *J Infect Dis.* (2014) 209:1963–71. 10.1093/infdis/jit842 24381206 PMC4038138

[18] Leanse LG Dong P Goh XS Lu M Cheng J Hooper DC Quinine enhances photo-inactivation of gram-negative bacteria. *J Infect Dis.* (2020) 221:618–26. 10.1093/infdis/jiz487 31565732 PMC7325614

[19] Barneck MD Rhodes NLR De La Presa M Allen JP Poursaid AE Nourian MM Violet 405-nm light: a novel therapeutic agent against common pathogenic bacteria. *J Surg Res.* (2016) 206:316–24. 10.1016/j.jss.2016.08.006 27884325

[20] Bumah VV Masson-Meyers DS Cashin S Enwemeka CS. Optimization of the antimicrobial effect of blue light on methicillin-resistant *Staphylococcus aureus* (MRSA) in vitro. *Lasers Surg Med.* (2015) 47:266–72. 25639752 10.1002/lsm.22327 PMC4834034

[21] dos Anjos C Sabino CP Bueris V Fernandes MR Pogliani FC Lincopan N Antimicrobial blue light inactivation of international clones of multidrug-resistant *Escherichia Coli* ST10, ST131 and ST648. *Photodiagnosis Photodyn Ther.* (2019) 27:51–3. 10.1016/j.pdpdt.2019.05.014 31100445

[22] Maclean M MacGregor SJ Anderson JG Woolsey GA Coia JE Hamilton K Environmental decontamination of a hospital isolation room using high-intensity narrow-spectrum light. *J Hosp Infect.* (2010) 76:247–51. 20864210 10.1016/j.jhin.2010.07.010

[23] Wozniak A Rapacka-Zdonczyk A Mutters NT Grinholc M. Antimicrobials are a photodynamic inactivation adjuvant for the eradication of extensively drug-resistant *Acinetobacter Baumannii*. *Front Microbiol.* (2019) 10:229. 10.3389/fmicb.2019.00229 30814989 PMC6381035

[24] Guffey JS Payne WC Motts SD Towery P Hobson T Harrell G Inactivation of *Salmonella* on tainted foods: using blue light to disinfect cucumbers and processed meat products. *Food Sci Nutr.* (2016) 4:878–87. 10.1002/fsn3.354 27826438 PMC5090652

[25] Yan Q Zhu H Sun X Dai T. Antimicrobial blue light inactivation of *Candida albicans* in an ex vivo model of keratitis. *Investig Ophthalmol Vis Sci.* (2018) 59:3661.

[26] Hoenes K Hess M Vatter P Spellerberg B Hessling M. 405 nm and 450 nm photoinactivation of *Saccharomyces cerevisiae*. *Eur J Microbiol Immunol.* (2018) 8:142–8. 10.1556/1886.2018.00023 30719331 PMC6348701

[27] Tomb RM Maclean M Coia JE Graham E McDonald M Atreya CD New proof-of-concept in viral inactivation: virucidal efficacy of 405 nm light against feline calicivirus as a model for norovirus decontamination. *Food Environ Virol.* (2017) 9:159–67. 10.1007/s12560-016-9275-z 28040848 PMC5429381

[28] Kingsley DH Perez-Perez RE Boyd G Sites J Niemira BA. Evaluation of 405-nm monochromatic light for inactivation of Tulane virus on blueberry surfaces. *J Appl Microbiol.* (2018) 124:1017–22. 10.1111/jam.13638 29144595

[29] Ettayebi K Crawford SE Murakami K Broughman JR Karandikar U Tenge VR Replication of noroviruses in stem cell-derived human enteroids. *Science.* (2016) 353:1387–93. 27562956 10.1126/science.aaf5211 PMC5305121

[30] de la Fuente-Núñez C Reffuveille F Fernández L Hancock REW. Bacterial biofilm development as a multicellular adaptation: antibiotic resistance and new therapeutic strategies. *Curr Opin Microbiol.* (2013) 16:580–9. 23880136 10.1016/j.mib.2013.06.013

[31] Wang Y Wu X Chen J Amin R Lu M Bhayana B Antimicrobial blue light inactivation of gram-negative pathogens in biofilms: in vitro and in vivo studies. *J Infect Dis.* (2016) 213:1380–7. 10.1093/infdis/jiw070 26908743 PMC4813746

[32] Ferrer-Espada R Wang Y Goh XS Dai T. Antimicrobial blue light inactivation of microbial isolates in biofilms. *Lasers Surg Med.* (2020) 52:472–8. 31536154 10.1002/lsm.23159 PMC7080594

[33] Liu X Chang Q Ferrer-Espada R Leanse LG Goh XS Wang X Photoinactivation of *Moraxella catarrhalis* using 405-nm blue light: implications for the treatment of otitis media. *Photochem Photobiol.* (2020) 96:611–7. 10.1111/php.13241 32105346 PMC10125262

[34] Ferrer-Espada R Liu X Goh XS Dai T. Antimicrobial blue light inactivation of polymicrobial biofilms. *Front Microbiol.* (2019) 10:721. 10.3389/fmicb.2019.00721 31024499 PMC6467927

[35] Angarano V Smet C Akkermans S Watt C Chieffi A Van Impe JFM. Visible light as an antimicrobial strategy for inactivation of *Pseudomonas fluorescens* and *Staphylococcus epidermidis* biofilms. *Antibiotics.* (2020) 9:171. 10.3390/antibiotics9040171 32290162 PMC7235755

[36] Martegani E Bolognese F Trivellin N Orlandi VT. Effect of blue light at 410 and 455 nm on *Pseudomonas aeruginosa* biofilm. *J Photochem Photobiol B Biol.* (2020) 204:111790. 10.1016/j.jphotobiol.2020.111790 31986339

[37] Koutchma T. UV light for processing foods. *Ozone.* (2008) 30:93–8.

[38] Bache SE Maclean M Macregor SJ Anderson JG Gettinby G Coia JE Clinical studies of the high-intensity narrow-spectrum light environmental decontamination system (HINS-light EDS), for continuous disinfection in the burn unit inpatient and outpatient settings. *Burns.* (2011) 38:69–76. 10.1016/j.burns.2011.03.008 22103991

[39] Kollef MH Torres A Shorr AF Martin-Loeches I Micek ST. Nosocomial infection. *Crit Care Med.* (2021) 49:169–87. 33438970 10.1097/CCM.0000000000004783

[40] Maclean M Booth MG Anderson JG MacGregor SJ Woolsey GA Coia JE Continuous decontamination of an intensive care isolation room during patient occupancy using 405-nm light technology. *J Infect Prevent.* (2013) 14:176–81.

[41] Ramakrishnan MK Sankar J Venkatraman J Ramesh J. Infections in burn patients – experience in a tertiary care hospital. *Burns.* (2006) 32:594–6. 10.1016/j.burns.2005.11.018 16713101

[42] Church D Elsayed S Reid O Winston B Lindsay R. Burn wound infection. *Clin Microbiol Rev.* (2006) 19:403–34. 16614255 10.1128/CMR.19.2.403-434.2006 PMC1471990

[43] Laciste MT De Luna MDG Tolosa NC Lu MC. Degradation of gaseous formaldehyde via visible light photocatalysis using multi-element doped titania nanoparticles. *Chemosphere.* (2017) 182:174–82. 10.1016/j.chemosphere.2017.05.022 28499178

[44] Zhu H Kochevar IE Behlau I Zhao J Wang F Wang Y Antimicrobial blue light therapy for infectious keratitis: ex vivo and in vivo studies. *Invest Ophthalmol Vis Sci.* (2017) 58:586–93. 10.1167/iovs.16-20272 28129422 PMC5283079

[45] Hoenes K Spellerberg B Hessling M. Enhancement of contact lens disinfection by combining disinfectant with visible light irradiation. *Int J Environ Res Public Health.* (2020) 17:6422. 10.3390/ijerph17176422 PMC7504152 32899295

